# Common Genomic and Proteomic Alterations Related to Disturbed Neural Oscillatory Activity in Schizophrenia

**DOI:** 10.3390/ijms26157514

**Published:** 2025-08-04

**Authors:** David Trombka, Oded Meiron

**Affiliations:** Brain and Memory Laboratory, Faculty of Education, Bar-Ilan University, Ramat Gan 5290002, Israel

**Keywords:** risk genes, quantitative EEG (qEEG), GABAergic interneurons, excitation/inhibition (E/I) balance

## Abstract

Schizophrenia (SZ) is a complex neuropsychiatric disorder characterized by heterogeneous symptoms, relatively poor clinical outcome, and widespread disruptions in neural connectivity and oscillatory dynamics. This article attempts to review current evidence linking genomic and proteomic alterations with aberrant neural oscillations observed in SZ, including aberrations in all oscillatory frequency bands obtained via human EEG. The numerous genes discussed are mainly involved in modulating synaptic transmission, synaptic function, interneuron excitability, and excitation/inhibition balance, thereby influencing the generation and synchronization of neural oscillations at specific frequency bands (e.g., gamma frequency band) critical for different cognitive, emotional, and perceptual processes in humans. The review highlights how polygenic influences and gene–circuit interactions underlie the neural oscillatory and connectivity abnormalities central to SZ pathophysiology, providing a framework for future research on common genetic-neural function interactions and on potential therapeutic interventions targeting local and global network-level neural dysfunction in SZ patients. As will be discussed, many of these genes affecting neural oscillations in SZ also affect other neurological disorders, ranging from autism to epilepsy. In time, it is hoped that future research will show why the same genetic anomaly leads to one illness in one person and to another illness in a different person.

## 1. Introduction

### Genes Affecting Neural Oscillations in Schizophrenia

SZ is a complex neuropsychiatric disorder that often begins in early adult life and continues throughout adulthood [1]. SZ (SZ) spectrum disorders have a complex and varied symptomology that may include hallucinations, paranoid ideation, delusions, and deficits in executive cognitive functions, including working memory, learning, verbal memory, executive attention, self-awareness, cognitive flexibility, problem-solving, and emotional regulation [2,3,4,5,6]. Researchers have shown that the pathological presentation of SZ is intertwined with impairments in connectivity within and between different cortical networks, including neurotransmitter-related synaptic dysfunctions [6,7,8,9,10]. Indeed, due to the heterogeneous symptomatology in SZ patient populations, its etiology cannot be localized to specific time periods or specific cortical region abnormalities that are common to all SZ phenotypes [11]. Friston (1998) posited the “disconnection hypothesis”, denoting impaired integration among distributed brain regions; specifically, aberrant connectivity within cortico-cortical and thalamocortical loops, as observed in several neuroimaging studies [12,13,14]. Therefore, high- and low-frequency neural oscillations (e.g., gamma and theta band frequency, respectively) obtained via human EEG analyses (e.g., qEEG) and interactions between different frequency bands in different mental states (rest versus stimuli-related activity) versus other psychiatric disorders may be critical to understand the complex and varied phenotypical presentation of SZ [15,16]. Accordingly, the main research goal in the current review will aim to highlight common and significant associations between abnormalities in neural oscillations and various genetic mutations found in SZ patient populations. Many of the genetic mutations detected in SZ patients are significantly associated with qEEG features under specific EEG electrodes denoting mean power and peak frequency under specific scalp electrodes (e.g., mean gamma power under frontal-midline electrodes), thus providing a genetic basis for neural-synchrony dynamics related to abnormal oscillatory brain activity (reduced frontal alpha power at rest) in SZ and other neuropsychiatric disorders [17,18,19,20]. Furthermore, recent research points to ensembles of genes (polygenicity) that influence the development of SZ-like symptoms through aberrant connectivity within specific neural frequency bands representing dysconnectivity among local and global brain networks, such as those within thalamocortical circuitries [21].

In regard to the nonsystematic review structure applied in the current paper, it is important to note that the review is written according to the PRISMA guidelines for a Scoping Review (https://www.prisma-statement.org/scoping, accessed on 29 June 2025), offering a comprehensive analysis of the existing literature within a field of study, and providing suggestions for future research.

## 2. Disturbed Neural Oscillations and Risk Genes in Schizophrenia

### 2.1. Gamma Oscillations

Neuronal network rhythmic-synchronization patterns reflected by changes in mean gamma power dynamics across brief periods in locally and globally distributed networks are often disrupted in SZ and seem to be essential for proper information processing, working memory item recognition, long-term memory recall, and accurate sensory perception [22]. Additionally, these deficits in information processing may include disruptions in astrocyte synapse-regulatory functions [23]. Disturbances in gamma oscillations (e.g., excessive frontal gamma power), associated with several genetic risk factors, are a characteristic hallmark of SZ [24,25]. Gamma “fast-wave” oscillations can be roughly separated into two frequency bands, 30–80 Hz and 80–250 Hz (the latter frequency range is known as high-band gamma). These gamma sub-frequencies are related to different cortical sensory-processing functions, as seen, for instance, in different gamma-activity patterns (spectral, temporal, functional) within the visual cortex [26]. GABAergic interneurons (parvalbumin and somatostatin) are critical for regulating timely gamma synchrony within cortical networks, as they regulate neuronal excitability and maintain neural excitation/inhibition (E/I) balance locally and modulate global ongoing slow-wave oscillations, such as theta-frequency oscillations [22,27].

E/I imbalance in SZ, particularly involving reduced inhibitory signaling from parvalbumin-positive (PV+) GABAergic interneurons and NMDA receptor hypofunction, disrupts the brain’s ability to regulate neural oscillations, especially, but not solely, in the gamma frequency range. This disruption can interfere with synchronized brain activity that underpins attention, working memory, and coherent top-down processing regulation. As a result, patients with NDMA hypofunction and abnormal gamma activity experience pronounce cognitive deficits (e.g., SZ patients), such as disorganized thinking and impaired working memory. Studies show reduced gamma-band auditory steady-state responses (ASSRs) and weaker functional connectivity in patients with SZ, correlating with symptom severity and supporting the use of oscillatory measures as biomarkers of cortical incoherence [28,29,30]. One of the most clinically significant consequences of E/I imbalance is the emergence of SZ positive symptoms, including hallucinations and delusions. The loss of inhibitory cortical control results in excessive, noisy cortical activity, particularly in sensory regions like the auditory cortex, making it difficult to distinguish internal from external stimuli. This hyperexcitable state is believed to underlie the spontaneous generation of voices (i.e., hearing voices) or distorted perceptions. As discussed by Jardri et al. (2016), aberrant excitation can give rise to hallucinatory experiences, especially when the auditory cortex exhibits abnormal gamma synchronization. These findings are consistent with research linking increased gamma activity with “noisy” left auditory cortex activity, and with severity of auditory verbal hallucinations [31,32]. As such, it is plausible to view E/I imbalance not only in its neural-activity regulatory-role in SZ, but also as a reflection of a global neural mechanism associated with molecular dysfunction and observable clinical symptoms.

### 2.2. KARs (Kainate-Type Glutamate Receptors)

Kainate-Type Glutamate Receptors (KARs) are ionotropic glutamate receptors and consist of five subunits, Gluk1–Gluk5, encoded via the *GRIK1* to *GRIK5* genes, each located on a different chromosome. Mouse models lacking the Gluk1 subunit in GABAergic interneuron KARs showed perturbed neurotransmission and aberrant spontaneous network activity in the hippocampus of neonatal mice. In adult male mice, the absence of GluK1 in GABAergic neurons led to stronger hippocampal gamma oscillations and enhanced theta–gamma cross-frequency coupling [33]. The Ser310Ala missense variant in *GRIK3* is associated with a higher risk and susceptibility to SZ. KARs influence gamma oscillations by modulating both excitatory and inhibitory synaptic transmission. They can facilitate synchronous rhythmic activity in neural networks, and their dysfunction can lead to abnormal oscillatory patterns in different brain circuitries. The ability of kainate to induce gamma oscillations emerges during early postnatal development, paralleling the maturation of GABAergic interneuron inhibitory circuits. It is worth noting that GABAergic influence shifts during development, from an initial excitatory (depolarizing) state to the mature inhibitory (hyperpolarizing) state [34,35,36]. Preclinical models show that selective KAR antagonists may have antipsychotic activity that can reduce behavioral abnormalities (e.g., hyperactivity, startle response deficits) in mice with NMDA receptor hypofunction [37,38,39,40].

### 2.3. KCN1 Gene and KCN2 Gene Encoding for the Potassium Channel Genes (Kv3.1 and Kv3.2)

The Kv3.1 and Kv3.2 voltage-gated potassium channels are part of the Kv3 family of voltage-gated potassium channels and play a pivotal role in the generation of gamma oscillations. These channels are expressed primarily in parvalbumin-positive (PV-INs) GABAergic interneurons. Interneuron activity is crucial for rapid neuronal firing and action potential repolarization, which is essential for synchronizing stimulus-related neuronal gamma-frequency oscillatory-reactivity across different cortical networks [41,42,43,44,45,46,47,48,49,50,51,52]. The genes encoding potassium channels Kv3.1 and Kv3.2 are *KCNC1* (11p15.1), and *KCNC2* (12q21.1), respectively [53,54,55]. Kv3.1 facilitates the rapid repolarization of neurons by enabling fast-spiking neuronal phenotypes, while Kv3.2 plays a similar role in neuronal excitability and function [48,51]. The Kv3.1 channels are critical for high-frequency rhythmic neuronal firing occurring across brief time intervals (100 to 300 ms). Mutations in this gene are primarily associated with progressive myoclonus epilepsy and ataxia (MEAK), but they also most likely contribute to psychiatric conditions like SZ [48]. Specific mutations, such as the recurrent heterozygous missense mutation c.959G>A (p.Arg320His) (substitution of arginine with histidine at position 320), lead to loss of Kv3.1 channel function, resulting in disrupted neuronal firing patterns and increased hyperpolarized potentials [42]. Mutations in *KCNC2* have been identified as causative factors for epilepsy and modifying factors for an array of neuropsychiatric disorders, including SZ. Functional studies show that certain *KCNC2* variants can lead to gain or loss of channel function, disrupting neural signaling and potentially contributing to SZ through altered neurotransmitter release and abnormal brain circuitry [46]. Genetic variants in *KCNC2* were linked to severe neurodevelopmental phenotypes, including epilepsy, speech disturbance, depression, and hyperactivity, though more research is required to show a direct link with SZ [49]. Regarding gamma oscillations, studies have shown that modulators of Kv3.1/3.2 channels in GABAergic interneurons can alter gamma power in SZ patients [45,46,48,51,56]. Significantly, Kv3.2 modulation using AUT00206, a Kv3.1/3.2 channel enhancer, restored striatal activation and gamma power regularity in SZ patients [56].

### 2.4. Neuregulin-1 (NRG-1) and ERBB4

SZ pathophysiology is closely associated with Neuregulin-1 proteins (NRG1, encoded by the *NRG1* gene (8p12) and its receptor ErbB4, encoded by the *ERBB4* gene (2q34). NRG1 proteins act as ligands for the ErbB family of receptor tyrosine kinases, transmitting signals that regulate cell differentiation, proliferation, growth, survival, and apoptosis. The four NRG1 proteins are also important for synapse formation and function, axon myelination, and excitatory and inhibitory neural connectivity balance. Genetic and molecular evidence revealed that their dysregulation can lead to SZ-related symptomology [57,58,59], especially due to non-coding *NRG1* SNPs in the 5′ upstream region, such as SNP8NRG221132 and SNP8NRG243177 [60], and *ERBB4* SNPs (e.g., rs7598440, rs839541), and due to splice-site mutations linked to risk for SZ. ERbB4 variants alter receptor splicing and downstream signaling leading to an overrepresentation of certain isoforms (especially JM-a and CYT-1) in the brain. These changes alter how ERbB4 receptors respond to neuregulin-1 and other ligands, shifting the balance of downstream signaling pathways (notably PI3K/Akt) and contributing to neurodevelopmental deficits and psychiatric disease risk, including SZ [61,62,63,64]. NRG1-ErbB4 modulates both glutamatergic and GABAergic transmission systems and controls the formation of dendritic spines and the translocation of cortical interneurons [65,66]. The NRG1 signaling pathway affects gamma oscillations through its interaction with ErbB4 receptors present in parvalbumin-positive interneurons [66,67,68,69]. The presence of NRG1 and ErbB4 polymorphisms are apparently linked to SZ risk. For example, certain polymorphisms of Erb4 alter the expression of certain isoforms, including CYT-1 [65,66,68]. These genetic variations may result in increased NRG1-ErbB4 signaling activity via phosphoinositide 3-kinase (PI3K)-dependent pathways [65,66,68]. Hyperactivation of the NRG1-Erb4 pathway operates through a postsynaptic density protein, PSD-95, facilitating ErbB4 activation, which in turn suppresses NMDA receptor function. Postmortem studies in SZ patients show increased ErbB4–PSD-95 interactions in the prefrontal cortex, which correlate with pronounced NMDA receptor hypofunction. These molecular alterations contribute to the disrupted glutamatergic signaling observed in SZ [67,69,70]. Furthermore, in murine models, the splice variant NRG1-beta, in contrast with NRG1-alpha, shows significant power augmentation in kainite-induced gamma oscillations due to its higher affinity with ErbB4 receptors [71].

NMDAR function disruption on GABAergic interneurons seems to be common in SZ and leads to downstream hyperactivation of glutamatergic and dopaminergic circuits [72,73]. The impaired PV-IN activity may disrupt synchronization of fast spiking networks that generate gamma oscillations. The NRG1-ErbB4 pathway inhibits Src kinase, which leads to decreased synaptic NMDAR activity and impaired long-term potentiation (LTP) activity in both hippocampal and cortical circuits [69]. ErbB4 is also responsible for controlling AMPA receptor (encoded by the *AMPA* family of genes) movement within cells as well as the development of dendritic spines. The disruption of NRG1-ErbB4 signaling leads to AMPA receptor destabilization and spine density reduction, which intensifies postsynaptic dysfunction [65,74,75,76]. Indeed, AMPA receptor dysfunction due to genomic or proteomic changes may lead to an inability to generate gamma oscillations [77,78]. *ERBB4* knockout animal (KO) model findings suggest SZ-like behavioral and neuroanatomical abnormalities, including impaired spatial learning and memory, hyperactivity, as well as disrupted cortical–hippocampal connectivity and increased dopamine levels in the striatum in affected animals [64,79,80]. Therefore, restoring ErbB4 receptor activity may improve neurotransmitter signaling in psychiatric disorders and may stabilize LTP activity [79]. Shi and Bergson (2020) have explored the possibility of restoring SZ-related cognitive and executive function deficits using gene therapy directed at NRG1–ErbB4 signaling in local circuit neurons in the PFC [81].

### 2.5. Neuronal Activity-Regulated Pentraxin, NARP

Neuronal Activity-Regulated Pentraxin (NARP) is important for regulating brain wave oscillations by modulating excitatory synaptic strength of parvalbumin-positive interneurons, which are important for E/I balance and neural oscillatory dynamics across the brain. This excitatory activity is regulated by the *NPTX2* gene (7q22.3). It is an early-immediate gene, responsible for clustering GluR4-containing AMPA receptors, thus enhancing excitatory drive onto PV interneurons for glutamate receptor-mediated excitation of PV-INS that promote gamma oscillations. PV-INs participate in gamma rhythms (30–80 Hz), critical for supporting E/I balance within local cortical networks. Due to its potentiation of the PV-IN excitatory input, NARP maintains an appropriate level of inhibitory tone to synchronize gamma activity [78,82]. Reduced *NPTX2* expression (lowered mRNA) in the DLPFC has been observed in SZ, leading to decreased excitatory drive onto parvalbumin interneurons and disturbed gamma activity [78]. PV-INs lacking NARP or having lower *NPTX2* mRNA levels exhibit reduced spontaneous firing rates (~50% decrease), which impairs their ability to generate timely rhythmic inhibition involving fast-rate synchronized post-synaptic inhibitory potentials. Interestingly, lowered NARP levels in DLPFC pyramidal neurons may lead to reduced clustering of AMPA receptors at excitatory synapses that increase parvalbumin interneuron inhibitory activity, thus contributing to lower excitatory drive to these targeted interconnected pyramidal neurons. In SZ, a reduced excitatory drive may result in lower GAD67 levels, leading to lower γ-aminobutyric acid (GABA) synthesis in parvalbumin interneurons and a reduced capacity to support the generation of timely gamma oscillations [78].

In individuals suffering psychosis-related symptoms (e.g., SZ spectrum disorders), lower *NPTX2* mRNA levels might also lead to less clustering of AMPAR at excitatory synapses of somatostatin interneurons [78]. Somatostatin-positive interneurons seem to complement the role of parvalbumin interneurons in modulating gamma synchrony (e.g., magnitude of absolute gamma power). Somatostatin-positive interneurons also modulate theta oscillations, and their dysfunction may disrupt gamma–theta coupling, worsening cross-network desynchronization [83]. Dienel et al. (2025) found that somatostatin interneurons in layers 2 and 3 of the DLPFC in individuals with SZ exhibited significantly lower levels of somatostatin and glutamic acid decarboxylase 67 (GAD67). A composite measure, termed the “presynaptic index of dendritic inhibition,” combining somatostatin mRNA and GAD67 mRNA levels, was lower in approximately 80% of individuals with SZ versus healthy controls. This reduction correlated with lower educational attainment, working memory impairments, and functional capacity deficits [84].

### 2.6. Phospholipase C-β1 (PLC-β1)

Phospholipase C-β1 (PLC-β1), encoded by the gene *PLCB1* (20p12.3), has been implicated in the regulation of gamma and beta oscillations, which are integral to sensorimotor gating and different cognitive processes related to information processing [85]. PLC-β1 knockout mice exhibit reduced power and phase synchrony of beta and gamma oscillations in response to auditory stimuli, correlating with behavioral deficits such as impaired prepulse inhibition PPI, a measure of sensorimotor gating deficits. PPI is a well-established endophenotype observed in SZ that aids in dissecting the genetic basis of the disorder [86]. These findings suggest that PLC-β1 dysfunction may contribute to high-frequency oscillatory abnormalities in SZ. Elevated ongoing beta oscillations at rest are also part of the oscillatory aberrations in PLC-β1 KO mice [85]. PLC-β1 deletion in the thalamic reticular nucleus (TRN) reduces neuronal excitability, leading to hypersynchrony in thalamocortical circuits. This manifests as spike-and-wave discharges (SWDs), resembling absence seizures, and disrupts feedforward inhibition critical for normal oscillatory rhythms [87]. Reduced PLC-β1 expression in the DLPFC of SZ patients is linked to working memory deficits. In mice, mPFC-specific PLC-β1 knockdown replicates these cognitive impairments, highlighting its role in maintaining prefrontal network synchrony to attain E/I balance [87,88]. The genetic mutation most directly linked to lowered PLC-β1 in SZ is a hemizygous deletion of the *PLCB1* gene [89,90]. Deletion of the *PLCB1* gene has been demonstrated in samples of SZ patients [89]. PLC-β1 integrates signaling from multiple targets implicated in SZ [91]. Udawela et al. (2017) argued that a lowered PLC-β1 in patients with SZ could affect multiple neurotransmitter pathways [92]. Most antipsychotic drugs and adjunctive treatments in SZ augment neurotransmitter receptor activity, as discussed in the next section; studies on SZ animal models or human brain tissue and PLC-β1 activity are noted [87,88,92,93].

### 2.7. Glutamate, Dopamine, Serotonin, Acetylcholine

The *GRM5* gene (11q14.2-q14.3) encodes a G protein-coupled receptor involved in glutamatergic neurotransmission, important for synaptic plasticity and neural network activity. KO mGluR5 (knockout mGlu receptor 5) mice showed aberrant synaptic plasticity, reduced PPI, memory deficits, and hyperactivity. mGlu5 KO mice show loss of NMDA receptor (NMDAR)-mediated components of hippocampal CA1 long-term potentiation (LTP) and reduced PPI, a hallmark of sensorimotor gating deficits seen in SZ. Deficits such as working memory are further exacerbated by NMDA antagonists like MK-801 (dizocilpine), suggesting mGlu5–NMDAR mutual and reciprocal interaction is critical [94,95,96]. In a significant display of the dual nature of SZ etiology, it was shown that when KO mice were exposed to housing conditions of environmental enrichment (EE), including sensory, cognitive, and social stimulation, these impairments were significantly attenuated. Furthermore, EE altered the behavioral response to the NMDA receptor antagonist MK-801 in these mice, displaying the strong interplay between mGlu5, NMDA receptor function, and environmental factors in SZ [97]. PLC-β1 is activated by mGluR1/5, which are G-coupled receptors. This activation generates inositol-1,4,5-trisphosphate (IP3) and diacylglycerol (DAG), modulating intracellular calcium release and protein kinase C (PKC) activity [98]. Reduced PLC-β1 expression impairs signaling pathways important for cognitive functions, including working memory and sensory processing, as shown in animal models with PLC-β1 knockdown or deletion. PLC-β1 is a critical part of mGluR-associated intracellular signaling cascades known to modulate NMDA receptor function and synaptic plasticity, but more direct evidence linking PLC-β1 deficiency to NMDA receptor impairment in SZ is needed. Nonetheless, disruptions in these pathways are thought to contribute to cognitive deficits and network dysfunction observed in SZ [87,88,93].

Dopamine D1 receptors (D1Rs) and dopamine D2 receptors (D2Rs) in the brain are coupled to PLC-β1 signaling pathways, participate in the regulation of intracellular calcium release, and synaptic plasticity. This coupling is essential for working memory [99]. PLC-β1 also facilitates serotonin signaling via the 5-HT2A and 2C receptors [100,101]. Reduced PLC-β1 impairs medial prefrontal cortex-dependent cognitive functions in animal models relevant to schizophrenia. While the precise effects on dopamine receptor signaling require further clarification, PLC-β1 deficiency contributes to multiple behavioral and cognitive endophenotypes linked to SZ [87,88,93]. *CHRM1* (11q13.4) is the gene encoding the M1 acetylcholine receptor; see below in the section on theta oscillations. *CHRM3* (1q43) is the gene encoding the M3 acetylcholine receptor. M1 KO mice exhibit increased locomotor activity and dopamine release, resembling SZ-like hyperdopaminergia [102,103]. Disrupted PLC-β1 signaling in the prefrontal cortex, notably in Gq-coupled M1/M3 muscarinic receptor pathways, can impair intracellular calcium and PKC-dependent signaling mechanisms critical for working memory and executive function. In mice, medial prefrontal cortex-specific knockdown of PLC-β1 reproduces schizophrenia-like endophenotypes, including working memory deficits. Further, theoretical models suggest that such deficits reflect broader disturbances in cholinergic-PLC signaling systems relevant to the symptoms of psychosis [88,104]. M1 mAChR activation via PLC-β1 has been implicated in the regulation of cortical network oscillations, particularly gamma oscillations. These oscillations are vital for mediating executive attention, and sensory integration during memory encoding and active information maintenance in working memory. If M1/PLC-β1 signaling is disrupted, it may contribute to the abnormal oscillatory activity (e.g., reduced gamma power) found to be related to cognitive and negative symptom severity in SZ. Both PLC-β1 KO and M1 mAChR KO may result in SZ-like phenotypes [87,104,105]. A subset of SZ patients (diagnosed with muscarinic receptor-deficit syndrome, MRDS) have markedly reduced M1 mAChR expression in the prefrontal cortex, further impairing PLC-β1-dependent signaling and potentially making them less responsive to M1-targeted therapies [106].

As discussed above, PPI deficits serve as a model for SZ sensorimotor gating impairment. In PLC-β1 knockout mice, both clozapine and olanzapine improve PPI, although they fail to normalize oscillatory deficits, indicating that PPI deficits are not directly related to cortical or thalamic oscillatory function [93,107,108]. McOmish et al. (2008) showed that clozapine, together with EE, can ameliorate some of the negative symptoms found in *PLCB1* knockout mice [107].

It should be noted that while oscillatory abnormalities are closely linked to many symptoms of SZ, not all behavioral or cognitive deficits are directly related to disrupted neural oscillations. As an example, olfactory dysfunction, often present in early-stage or high-risk individuals, is more strongly associated with structural irregularities in the orbitofrontal cortex and olfactory bulb than with altered oscillations [109]. Furthermore, smooth pursuit eye movement (SPEM) deficits reflect dysfunction in the frontal eye fields and cerebellum rather than abnormalities in oscillatory activity [110]. Dissociation between neural oscillations and clinical improvement is seen in PPI, a measure of sensorimotor gating. Antipsychotics such as clozapine and olanzapine enhance PPI without normalizing gamma-band deficits, pointing to the involvement of brainstem and mesolimbic circuits in mediating gating functions independently of cortical rhythms [111]. Therefore, while abnormal oscillations are a major component of SZ pathophysiology, several symptomatic realms are more readily explained by structural or synaptic activity dysfunctions within frontomesolimbic neural circuitries. Negative symptoms like avolition and flattened affect are linked with dopaminergic and limbic system dysfunction, especially within the ventral striatum [112].

### 2.8. ARX

The *ARX* gene (Xp21.3) may be important in the prenatal etiology of SZ as it is critical for the proper development and function of PV GABAergic interneurons. Immunological challenge, such as that of the rodent model Maternal Immune Activation (MIA), cause in adult offspring reduced auditory-evoked gamma and theta oscillatory power as well as reduced PV protein levels. Furthermore, the *ARX* gene is reduced in the forebrain of MIA-exposed mice. In humans, a novel missense mutation in a SZ patient was identified. Nakamura et al. (2019) conjecture that MIA may influence *ARX* expression and induce GABAergic dysfunction. They further conclude that *ARX* variants may play a role in the prenatal etiology of SZ [113].

### 2.9. DTNBP1

The *DTNBP1* gene (dystrobrevin binding protein 1, 6p22.3) encodes the protein dysbindin, which plays a role in intracellular protein trafficking and synaptic function. Within neurons, it is involved in regulating the trafficking of synaptic vesicle proteins and the surface expression of neurotransmitter receptors.

Carlson et al. (2011) found that mice lacking sufficient dysbindin-1 exhibited significant deficits in fast-phasic inhibition, particularly involving parvalbumin-positive interneurons, which are essential for the generation of gamma oscillations in the brain. These mice showed reduced gamma-band activity in response to auditory stimuli. The study further demonstrated that these neural circuit disruptions led to behavioral deficits in sensory processing and memory, which are also characteristic of SZ. It seems, then, that loss of dysbindin-1 impairs the ability of neuronal networks to synchronize at high frequencies, providing a mechanistic link between a genetic risk factor for SZ and the neural oscillatory and cognitive abnormalities seen in the disorder [114].

### 2.10. PVALB

The PVALB gene (22q12) in humans encodes parvalbumin, a high-affinity calcium-binding protein expressed predominantly in a specific subset of fast-spiking GABAergic interneurons in the brain. Deficits in PV+ interneurons—such as reduced PVALB expression or impaired function—are consistently observed in SZ and are directly linked to disturbances in gamma oscillatory activity [115].

### 2.11. GAD1

The *GAD1* gene (2q31.1) encodes the enzyme glutamate decarboxylase 67 (GAD67), which catalyzes the production of the inhibitory neurotransmitter gamma-aminobutyric acid (GABA) from glutamate. Reduced *GAD1* expression weakens gamma band synchrony by impairing GABAergic neurotransmission, which in turn disrupts the inhibitory control required for proper neural synchrony and oscillatory rhythms in the cortex [116].

Animal studies show that *GAD1* knockout can result in increased spike-wave discharges, which are abnormal oscillatory events often in the lower frequency (delta/theta) range, particularly in thalamocortical circuits [117,118].

### 2.12. BDNF (Brain Derived Neurotrophic Factor)

*BDNF* (11p.14.1) encodes a protein vital for the survival, growth, and differentiation of neurons in the brain and spinal cord. Research has shown that the *BDNF* Val66Met genotype affects neural oscillatory activity. Studies using EEG and MEG have demonstrated that Met allele carriers exhibit differences in oscillatory power and connectivity compared to Val homozygotes. These differences are observed in various frequency bands, including alpha and gamma, and are associated with altered cortical excitability and functional connectivity [119,120].

The *BDNF* Val66/Met polymorphism is associated with cognitive deficits, altered functional connectivity, changes in neural oscillatory patterns, and altered mismatch negativity (MMN) affecting working memory in SZ patients [121,122].

### 2.13. Beta Oscillations

Beta oscillations (12–30 Hz) are involved in sensory and motor processing; abnormalities in beta oscillations have been observed in SZ and are linked to specific genetic factors. As mentioned above, in addition to its role in gamma oscillations, PLC-β1 also influences beta oscillations. PLC-β1 knockout mice exhibit elevated ongoing beta oscillations, which are correlated with behavioral deficits in a mouse model of SZ [85,88].

### 2.14. Auditory Steady-State Responses (ASSRs)

Auditory Steady-State Responses (ASSRs) are brain oscillations evoked by rapid and repetitive auditory stimuli (for example, 40 Hz auditory-click frequency). They reflect synchronized activity in thalamocortical circuits involving pyramidal neurons and parvalbumin GABAergic interneurons and are used to assess sensory reactivity in the auditory cortex and connected neural circuit integrity. As noted above, GABAergic interneurons, regulating E/I balance, play a critical role in generating both gamma and beta oscillations. In SZ, impaired interneuron function leads to poor E/I balance, resulting in abnormal oscillatory activity [123,124,125,126]. According to computational studies, beta and gamma rhythms may reflect distinct aspects of neuronal population synchronization during sensory processing. Although ongoing input usually prompts the formation of neuronal assemblies at gamma rhythms, the emergence of a beta rhythm changes these assemblies into new patterns via a rebound from inhibition [125,126]. In addition, beta and gamma oscillations could underlie the flow of information in opposite directions, that is, forward vs. backward [127,128,129]. Along the lines of predictive coding, this suggests that prediction errors could be propagated in a feed-forward manner, mainly using the gamma frequency channel, whereas predictions (and their revisions) could be transmitted [130,131,132,133]. The 40 Hz ASSR represents activation of a specific neural circuit tuned to gamma frequencies, but this circuit can also be engaged by certain beta-band stimuli [134]. The *HCN1* gene (discussed below) has been primarily linked to reduced gamma power and coherence in ASSRs and to altered theta oscillations [135,136].

The “beat-skipping” rhythm refers to an aberrant auditory gamma-band response in which, during 40 Hz stimulation, the brain exhibits a resonance at approximately 20 Hz, effectively skipping every other gamma cycle. This shift, simulated in computational models of prolonged GABAergic inhibition, reflects impaired gamma synchronization—a core signature of schizophrenia—and indicates compensation via beta-frequency activity, which is less effective for supporting the high-resolution temporal processing required for perception and cognition. Clinically, this disrupted neural entrainment is reflected in schizophrenia patients as reduced 40 Hz power and phase-locking, along with increased beta-band activity during gamma auditory steady-state response (ASSR) tasks [137,138,139,140]. Such abnormalities have been strongly linked to GABAergic layer-specific deficits in the auditory and prefrontal cortices, supported by evidence showing altered neural synchrony and ASSR responses in both SZ and related disorders [138,139,140]. These aberrations are tied to GABAergic and NMDA dysfunction and may underlie symptoms like hallucinations and disorganized thought, as gamma oscillations are crucial for cognitive processes like attention and working memory. The investigation of NMDA receptor partial agonists (D-serine and D-cycloserine) as an adjunctive treatment to specific serotonergic agonists (e.g., serotonergic psychedelics) in SZ is ongoing, and it was shown to modulate neuroplasticity and induce procognitive effects via 5HT2AR–NMDAR interactions. These serotonergic-glutamatergic circuitry interactions were shown to modulate neuronal excitability in the prefrontal cortex and represent a target for “novel antipsychotics” in the treatment of SZ [141,142].

### 2.15. GRIN2 and AKAP11

*GRIN2A* (16p13.2), encoding the GluN2A subunit of the NMDA receptor, and *AKAP11* (13q14.11), encoding the A-kinase anchoring protein, are high-risk genetic factors for SZ, with their mutations contributing to disruptions in neural signaling, synaptic plasticity, corticolimbic and thalamocortical circuit dysfunction, and neural oscillations across different frequency bands [143,144,145,146,147,148,149,150,151,152,153]. The GluN2A subunit of NMDA receptors is critical for synaptic plasticity and excitatory neurotransmission, and SZ-associated variants of *GRIN2A* result in loss-of-function mutations, leading to reduced NMDA receptor activity, a well-studied excitatory postsynaptic mechanism underlying SZ pathology. Interestingly, GluN2A shows two distinct types of genetic variants. One type includes rare variants affecting protein synthesis, such as protein-truncating and missense variants. The second type, the more common mutations, do not affect the actual protein but do alter gene expression and protein levels through non-coding or regulatory mutations [146,147,148,149,154]. Mouse models of *GRIN2A* mutants show altered cortical–hippocampal–striatal activity, elevated dopamine signaling, elevated resting gamma power, and disrupted gamma band ASSRs at 40–50 Hz and altered mismatch negativity (MMN) ERP responses [6,143,144,145,148,149].

*GRIN2A* expression begins postnatally and increases through adolescence, aligning with the typical onset of SZ during early adulthood [146,148,152,154]. *GRIN2A* polymorphisms (rs7206256, rs11644461) are associated with early-onset (before age 18) SZ [152]. Targeting NMDA receptor hypofunction linked to *GRIN2A* could alleviate cognitive and negative symptoms of SZ [146,148,154,155,156]. Studies have investigated the relationship between *GRIN2* and gamma oscillations, but the literature also addresses specific *GRIN2* mutations and beta activity as potential biomarkers in SZ [144,145,148,149]. It is important to call attention to the fact that *GRIN2A* mutants exhibit increased sleep spindle density, while *AKAP11* mutant mice show reduced sleep spindle density [144].

Sleep spindle density refers to the number of sleep spindles, which are brief bursts of brain activity, occurring per unit of time during stage 2 non-REM sleep. These spindles typically oscillate in the sigma frequency range (approximately 11–16 Hz) and are generated through coordinated interactions within thalamocortical networks. They play a crucial role in memory consolidation, synaptic plasticity, and sensory gating during sleep [157,158,159]. In SZ, sleep spindle abnormalities have been observed and are considered a biomarker of disrupted thalamocortical function, which is a fundamental feature of the disorder [157,158,159]. The observation that *GRIN2A* mutants exhibit increased spindle density while *AKAP11* mutants show reduced spindle density is significant because it underscores the diverse neurophysiological effects that different SZ-related genes can exert on neural oscillations spanning over alpha-to-beta frequency bands [144]. This divergence suggests that not all genetic pathways create the same type of oscillatory disruption, which has implications for personalized medicine and in understanding distinct clinical subtypes within the disorder, emphasizing the heterogeneous nature of SZ.

*AKAP11* encodes a scaffolding protein that anchors protein kinase A (PKA) and interacts with glycogen synthase kinase 3 beta (GSK3β), both involved in neuronal plasticity and signaling. Loss-of-function mutations in *AKAP11* disrupt these pathways, resulting in elevated resting gamma power, contributing to SZ risk and bipolar disorder-related abnormal neural oscillatory activity [144,160,161]. *AKAP11* knockout elevates synaptic PKA levels, leading to impaired synaptic plasticity, disrupting cortico-striatal-thalamic circuits, suggesting that beta-gamma synchronization is altered globally across thalamocortical networks in SZ. These findings align with models implicating corticolimbic and thalamocortical circuit dysfunction as druggable targets in SZ [143,144,150,151,160,161].

### 2.16. GABRA2

The *GABRA2* gene (4p12) encodes the α2 subunit of the GABAA receptor, a ligand-gated chloride channel that is primarily associated with modulating beta oscillations and is linked to alcohol dependence, epilepsy, and other neuropsychiatric conditions. Tissue-specific expression analyses suggest that *GABRA2* may mediate its effects through GABAergic interneuron activity [162,163,164,165,166,167]. *GABRA2* polymorphisms are associated with SZ risk and related endophenotypes, including altered GABAergic signaling and associated cognitive deficits, with brain region-specific expression deficits in SZ, bipolar disorder, and depression [167]. Postmortem studies report reduced *GABRA2* mRNA in the superior temporal gyrus (STG) and midbrain of SZ patients, suggesting region-specific GABA-A receptor dysfunction [165]. Conversely, increased *GABRA2* protein/mRNA has been observed in the lateral cerebellum of SZ patients, highlighting brain-region variability in GABAergic pathology [167]. Due to the extensive effect of *GABRA2* on oscillatory synchronization, alpha and gamma oscillations may be affected as well if the gene is downregulated [163].

### 2.17. Theta Oscillations

#### 2.17.1. *PLCB1* Gene (PLC-β1)

See above concerning beta and gamma oscillations. Regarding theta oscillations, *PLCB1* is involved in cholinergic (muscarinic) theta generation, specifically for attention and sensory processing, but not motor activity [168,169].

#### 2.17.2. *ZNF804A*

Theta oscillations (4–7 Hz) play a critical role in cognitive processes such as memory consolidation, attention, and memory retention processes related to hippocampal–prefrontal connectivity. In SZ, abnormalities in neural theta rhythms are linked to genetic risk factors such as *ZNF804A*, *GRIA1*, and *COMT*, which modulate synaptic plasticity, dopaminergic signaling, and neural synchrony between different neural networks [170,171,172,173,174,175,176]. The SZ-associated single-nucleotide polymorphism rs1344706 exerts its effects during the second trimester of fetal development. It is located on an intron of the *ZNF804A* gene (2q32.1), and its T allele is associated with decreased expression of *ZNF804* [173,174,175,176]. Carriers of the *ZNF804A* risk allele have a higher auditory-evoked P300 component amplitudes than non-carriers; increased P300 auditory-evoked responses represent a putative endophenotype of SZ. Theta oscillations normally coordinate hippocampal–prefrontal activity, but *ZNF804A* risk-homozygotes exhibit impaired episodic, spatial, and working memory in SZ patients, correlating with theta-driven hippocampal–prefrontal dysconnectivity in SZ versus healthy controls [173,174,175,176]. However, interestingly, carriers of this gene show low-to-normal IQ levels despite their observed working memory dysfunction [170]. Risk homozygotes show decreased theta band activity in the hippocampus with hyperconnectivity between the hippocampus and the superior frontal gyrus during rest, although hypoconnectivity in these networks was observed during working memory tasks [171,174,175,176]. The risk allele is associated with abnormal synaptic function and altered expression of genes associated with synaptic connectivity and neural plasticity, such as *BDNF* and *GRIN2A* [173]. Furthermore, Voineskos et al. (2011) propose a link between the risk variant ZNF804A and structural changes in memory-critical brain regions, such as the cingulate cortex and temporal cortices. Individuals homozygous for the risk variant rs1344706 displayed reduced cortical gray matter thickness in the anterior and posterior cingulate cortices and the superior temporal gyrus [176].

#### 2.17.3. *COMT*

The *COMT* gene (22q11.2) encodes an enzyme that breaks down catecholamines, especially dopamine. The interplay between *COMT* genotype, dopamine availability, and NMDAR function has been implicated in various neuropsychiatric conditions, including SZ [177,178,179,180]. Optimal D1 dopamine receptor activation enhances NMDAR currents via cAMP-PKA pathways. *COMT* Val/Val homozygotes (high *COMT* activity) exhibit lower dopamine levels, impairing D1 receptor signaling. Low dopamine levels in Val/Val carriers reduce NMDAR-mediated synaptic plasticity, contributing to hypofunction. Furthermore, Val/Val carriers show significant upregulation of glutamic acid decarboxylase 67 (GAD67) and glutamate NMDA receptor subunit epsilon-1 (GluN2A) mRNA levels [177,178]. The *COMT* Val158Met polymorphism alters prefrontal dopamine levels, while Val/Val homozygotes show faster dopamine degradation, reduced delta, theta, and beta activity, and slower alpha activity in both wakefulness and sleep, while Met/Met carriers exhibit elevated theta power during rest linked to hyperdopaminergia [179,180,181,182,183]. Steiner et al. (2019) showed that higher Schizotypal Personality Questionnaire (SPQ) scores were registered for both the homozygous Val/Val and Met/Met genotypes than for the Val/Met genotype and that complex neural oscillatory differences are seen between the Val/Val SZ risk individuals and actual schizophrenic individuals [184].

Glutamatergic dysregulation observed in certain *COMT* and *ZNF804A* risk alleles seems to directly affect NMDA receptor hypofunction observed in SZ. *ZNF804A* risk variants impair hippocampal–prefrontal theta coherence [170], while *COMT* Val alleles exacerbate NMDA-driven cortical noise, reduced signal-to-noise ratio, and inefficient prefrontal function [185,186]. Disruptions in theta-gamma cross-frequency coupling have been linked to working memory and sensory integration deficits in schizophrenia, although some studies report intact coupling in specific sensory domains. Independent evidence also implicates *COMT* polymorphisms in working memory impairments through their effects on prefrontal dopamine signaling [15,187,188]. The *COMT* genotype can influence response to antipsychotic medications such as risperidone, olanzapine, and clozapine. For example, patients with the Met/Met genotype may show greater improvement in executive function and positive symptoms compared to Val/Val or Val/Met genotypes when treated with antipsychotic medications [189,190].

#### 2.17.4. *KCNJ*

The *KCNJ* genes 3,6,9,5 encode the GIRK G-protein potassium channels 1,2,3,4, respectively. Each gene is located on a different chromosome. Dysregulation of GIRK channels has been linked to the pathophysiology of SZ, potentially contributing to abnormal theta and gamma oscillations in the hippocampal and thalamic circuits [191,192,193].

#### 2.17.5. *CHRM1*

*CHRM1* (11q13) modulates hippocampal and cortical circuits, supporting cognitive functions that depend on theta oscillations, such as working memory and attention, and is crucial for generating the essential hippocampal theta oscillations characteristic of REM sleep. Double knockout (DKO) mice lacking *CHRM1* and *CHRM3* show an abolishment of the REM stage of sleep [194].

### 2.18. Delta Oscillations

Delta oscillations (1–4 Hz) are central to sleep regulation and varied cognitive processes [195,196].Abnormal delta activity in SZ is linked to genetic risk factors affecting thalamocortical circuits, NMDA receptor signaling, and dopaminergic pathways. Reduced delta activity in SZ during slow-wave sleep (SWS) is tied to impaired memory consolidation and synaptic downscaling [195,196]. Thalamic reticular nucleus (TRN, also known as nRT) parvalbumin (PV) neurons regulate thalamocortical delta rhythms during sleep and wakefulness. As such, TRN-PV activity alterations result in abnormal delta and gamma oscillations at rest, which seems to underly SZ pathophysiology in both local and global brain circuitries [197]. NMDAR hypofunction at thalamocortical synapses disrupts glutamatergic signaling, leading to hyperpolarization of TRN-PV neurons. This hyperpolarization activates T-type Ca^2+^ channels, generating increased delta activity during wakefulness. In SZ, dopamine 2 (D2) receptor hyperactivation can intensify, via GIRK potassium channels, which leads to thalamic hyperpolarization, further promoting abnormal delta rhythm dominance [198].

#### 2.18.1. *GRIN2A* (GluN2A NMDA Receptor Subunit)

*GRIN2A* (16p13.2) is discussed above. Regarding theta oscillations, it regulates NMDA receptor-dependent synaptic plasticity in thalamocortical circuits [148,198]; loss-of-function mutations may lead to low-frequency paroxysmal waveforms and altered delta frequency oscillations during sleep in *GRIN2A* knockout mice [156,199]. Because *GRIN2A* encodes a key NMDA receptor subunit, several clinical trials have explored NMDA receptor modulators as adjunctive treatments for SZ [146].

#### 2.18.2. *CACNA1I* (Voltage-Gated Calcium Channel)

*CACNA1I* (22q13.1), encodes the CaV3.3 α1I subunit of T-type calcium channels, critical for low-threshold calcium spikes (LTS) in TRN neurons. This gene plays a role in neuronal excitability and has been implicated in neuropsychiatric disorders, including SZ. SZ-associated variants (e.g., R1346H) reduce CaV3.3 membrane expression, CaV3.3 protein levels, and current density, impairing rebound bursting in TRN neurons, potentially disrupting thalamocortical synchrony, and amplifying delta oscillations during wakefulness [200,201]. Studies in animal models with *CACNA1I* mutations demonstrate that correcting sleep spindle deficits by targeting CaV3.3 or TRN function could be a novel therapeutic strategy for SZ, especially for cognitive and sleep-related symptoms [201,202]. Ferrarelli (2021) discusses the literature indicating that the antipsychotic clozapine, as well as neuromodulator techniques such as ECT and TMS, may enhance GABA-mediated inhibition and, thus, improve sleep [202].

#### 2.18.3. *SCN1A*

*SCN1A* (2q24.3) encodes the Nav1.1 sodium channel. Mutations in this gene are known to impair the firing of GABAergic interneurons, particularly parvalbumin-positive (PV+) neurons, which are critical for the generation and timing of brain oscillations, especially delta and gamma rhythms [138,203,204,205]. These impairments in GABAergic neurotransmission are associated with cognitive impairment and altered oscillatory dynamics in both epilepsy and several psychiatric disorders. Interestingly, the p.L1649Q mutation in *SCN1A* was found to increase delta oscillatory power in human neural circuits during resting-state conditions and in response to synaptic input patterns mimicking in vivo cortical up/down states [203,204].

#### 2.18.4. *HCN1*

The *HCN1* (hyperpolarization-activated cyclic nucleotide–gated channel 1) gene (5p12) encodes an ion channel that contributes to the hyperpolarization-activated current (Ih), which regulates neuronal excitability and neural rhythmic activity in the brain. It is unique since it is activated by hyperpolarization, unlike other ion channels. *HCN1*-encoded channels are highly expressed in the dendrites of pyramidal neurons in the cortex and hippocampus, and in the axons of parvalbumin-positive interneurons, where they regulate both excitatory and inhibitory synaptic integration [135,206,207]. Growing evidence shows that changes in *HCN1* expression or kinetics in cortical layer V pyramidal cells increase network gain and responsiveness to delta-frequency inputs, paralleling the increased delta power seen in SZ versus healthy controls in resting-state EEG in the eyes closed condition [206,208,209,210,211,212]. Targeting HCN1 channels could influence neuronal oscillations and cognitive processes relevant to SZ pathology, particularly cognitive impairment associated with the illness severity [211]. Experimental invasive neuromodulation techniques, such as piezoelectric silk-based implants, have been explored in animal models to restore normal firing patterns in brain regions with *HCN1* dysregulation, but these approaches obviously remain far from clinical application [212].

#### 2.18.5. *KCNB1*

The *KCNB1* gene (20q13.13) is also discussed above. It encodes the Kv2.1 potassium channel, a voltage-gated K^+^ channel important for regulating neuronal excitability, action potential repolarization, and neural firing patterns. The deletion of Kv2.1 leads to neuronal hyperexcitability and related cognitive symptoms [213]. Layer 5 pyramidal cell (L5PC) networks are capable of intrinsically generating delta rhythms through the action of small-conductance calcium-activated potassium (SK) channels. These SK channels mediate the afterhyperpolarization (AHP) that follows bursts of action potentials, shaping the timing and stability of synchronized rhythmic firing. When calcium enters the neuron, primarily through NMDA receptor activation during synaptic transmission, SK channels are activated, leading to AHP that regulates excitability and supports the pacing of ongoing slow oscillations such as delta rhythms [214,215]. Prolonged AHP in PV+ interneurons reduces their ability to fire at high frequencies, impairing gamma rhythm generation [214,215,216]. KCNB1 variants are known to disrupt normal potassium currents, leading to altered network excitability. When these channels are dysfunctional, the delicate balance of ionic conductance that underlies the generation and stability of delta rhythms is disturbed. This can result in increased delta power and impaired neural-oscillatory rhythm-stability, which are brain-state features observed in some neurodevelopmental and neuropsychiatric disorders, including SZ [217].

#### 2.18.6. *DISC1*

*DISC1* (1q42.2) regulates neurodevelopmental processes such as synaptic adhesion and neurogenesis and plays a role in glutamatergic signaling, modulating glutamate release. Lymphocyte-derived or lymphoblastoid-derived *DISC1* gene expression has shown variability in psychiatric diseases, such as SZ and schizoaffective disorder [218], and is considered a reliable model for SZ endophenotype research [219,220]. *DISC1* expression has been shown to have great heritable variation within its promoter region and is also influenced through other gene regions and through other genomic regions (3p25, 7p15) [219]. Narayanan et al. (2015) identified *DISC1* as a key candidate risk gene mediating delta and theta EEG oscillatory abnormalities in SZ and psychotic bipolar disorder. Interestingly, the authors showed that theta abnormalities mediated by *DISC1*-related pathways differentiate SZ from psychotic bipolar disorder, showing distinct genetic and molecular pathways involving glutamatergic signaling and synaptic adhesion in patients with increased psychosis-related symptoms [220]. *DISC1* mutations or downregulation have also been implicated in impaired gamma oscillations in the hippocampus and PFC due to a reduced number of fast-spiking PV interneurons [221,222].

#### 2.18.7. Synaptic Gene—*NRGN*

NRGN (11q24.2) encodes neurogranin, a postsynaptic protein involved in synaptic plasticity and calcium signaling. The single-nucleotide polymorphism rs12807809 in *NRGN* is significantly associated with SZ, particularly in Caucasian populations [223,224]. Delta neural oscillations may be affected by the rs12807809 variant by impairing calcium signaling in GABAergic interneurons and by altering synaptic plasticity in dopamine-modulated circuits, which could affect delta activity [203,204]. *NRGN* variants may serve as biomarkers for SZ risk, cognitive dysfunction, and brain structural changes [225,226,227]. Hwang et al. show that components of the calcium/calmodulin-dependent signaling cascade, particularly the neurogranin-protein phosphatase-NMDAR axis, provide potential targets for the therapeutic remedy of cognitive impairment in SZ and other psychiatric conditions such as Alzheimer’s Disease [224]. Neurogranin plays a crucial role in synaptic plasticity, synaptic regeneration, and long-term potentiation (LTP) via calcium- and calmodulin-signaling pathways. Reduced expression of neurogranin has been observed postmortem in the prefrontal cortex in patients with SZ [223,224]. Beta oscillations may be affected by the rs12807809 variant by impairing calcium signaling in GABAergic interneurons, and by altering synaptic plasticity in dopamine-modulated circuits, which could also affect delta oscillations [227]. *NRGN* variants may serve as biomarkers for SZ risk, cognitive dysfunction, and brain structural changes [225].

### 2.19. Alpha Oscillations

Several genes at the short arm of chromosome 3 are associated with alterations in alpha power, suggesting a polygenic basis underlying abnormal alpha activity in humans. Genes such as *GNL3* and *ITIH4* in specific brain regions correlate with ongoing alpha activity [163]. Additionally, although *GABRA2* is primarily associated with beta oscillations, its role in the broader context of neural oscillations suggests potential indirect effects on alpha oscillations.

#### 2.19.1. *GNL3*

*GNL3* (3p21.1) sits in a genomic region strongly linked through GWAS to SZ and bipolar disorder. Risk alleles at this locus correlate with altered *GNL3* mRNA expression in the dorsolateral prefrontal cortex (DLPFC). Mendelian randomization and functional studies show that *GNL3* variants (rs10865973, rs12635140) regulate neuronal proliferation and dendritic spine density, mirroring synaptic deficits observed in SZ [163,228,229]. *GNL3* knockdown or overexpression disrupts neuronal differentiation in human neural progenitor cells and forebrain organoids, suggesting its role in neurodevelopmental pathways implicated in SZ [229,230]. *GNL3* and genes in the vicinity of 3p21.1 are known to influence alpha oscillations and are associated with clinical risk for SZ and bipolar disorder, suggesting a mechanistic link between *GNL3*, abnormal alpha rhythms, and risk for SZ [163].

#### 2.19.2. *ITIH4*

*ITIH4* (3p21.1) is co-localized with *GNL3* at the locus and is pleiotropically associated with SZ and cortical surface area (SA) alterations [231,232]. The *ITIH4* locus is enriched for SZ risk SNPs that correlate with mRNA expression changes in the DLPFC [230,231,232]. While *ITIH4* knockout mice show no behavioral changes, broader *ITIH* family disruptions (e.g., Ambp/bikunin—genes necessary for functional *ITIH1* and *ITIH3* complexes) alter anxiety and social behaviors, hinting towards their indirect roles in generating neuropsychiatric symptoms and affective dysregulation in humans [231]. *ITIH4* levels have been observed to be significantly decreased in the serum of SZ patients, such that it could serve as a potential clinical biomarker for diagnosis or for disease monitoring in SZ patients over time [232]. *ITIH4* is an SZ risk gene, and its impact on functional alpha activity may link it to disrupted attention, sensory processing, and executive attention deficits in SZ. SNPs in the gene locus region have been shown to alter gene expression, leading to altered alpha oscillations in the frontal cortex, observed in SZ patients [163,232].

## 3. Conclusions

This review attempts to outline the current research on the genetic underpinnings of neural oscillatory abnormalities in SZ. It is not exhaustive, as the complexity of the phenomena would require a more elaborate review analyzing genetic mutations and associated abnormal brain connectivity directly from a large population of SZ patients, from prodromal stages to disease onset, to treatment response. However, the current research review sheds light on the central genomic and proteomic factors causing neural oscillatory aberrations in SZ (See Table 1). The article highlighted the widespread dysconnectivity within and between different thalamocortical subnetworks, stressing the functional role of fast-spiking GABAergic interneurons and glutamatergic circuits in SZ pathophysiology, and the excitatory/inhibitory roles of these circuits in the generation and regulation of neural oscillation synchrony related to cross-network and post-synaptic neural-network dynamics in healthy humans. See Supplementary Files for illustrations of key signaling pathways associated with altered oscillations in SZ.

Finally, our findings reflect the polygenic nature of SZ, noting that ensembles of risk genes collectively contribute to abnormal neural oscillatory patterns obtained via scalp EEG under specific electrodes, and across the entire scalp. The interplay between genetic mutations and oscillatory dysfunction (e.g., excessive global gamma power) is supported by both human EEG studies, animal models, and computer modeling, demonstrating that alterations in genes regulating ion channels, glutamate and GABA receptors, E/I balance, and synaptic scaffolding proteins are highly related to SZ psychopathology and neural network dysconnectivity. Furthermore, there are potential avenues for non-invasive therapeutic interventions targeting specific cortical areas to restore normal oscillatory function in neurological disorders [6,70,233,234], and particularly in SZ patients. Additionally, it is important to note heterogeneity in SZ patient populations and include factors such as age, gender, and duration of illness, as they could impact the magnitude of neural activity disruption as well [235].

## 4. Future Directions

In response to recent findings showing that elevated resting gamma power in SZ is related to SZ symptom severity and that E/I imbalances are associated with global GABAergic neurotransmission dysfunction in SZ [6], we propose to routinely examine resting global gamma power differences and changes over time during “eyes-open” conditions in younger at-risk individuals versus healthy controls (e.g., prodromal SZ patients and in their first-degree healthy relatives and non-relatives). It is recommended to evaluate resting gamma power before and after the onset of the disease in individuals with genetic risk factors versus controls, to support resting gamma power as a predictive biomarker for disease onset and progression in high-risk individuals for psychosis (i.e., psychosis proneness). In support, gamma-power alterations are highly associated with risk genes that collectively contribute to abnormal oscillatory patterns and altered neural connectivity in thalamocortical–limbic circuitries in SZ and could be a reliable resting brain-state endophenotype that can distinguish high-risk from low-risk individuals at prodromal stages of the disease. However, a recent systematic review and meta-analysis indicated the heterogeneity of resting-state gamma power alterations in patients with SZ [235]. Resting gamma power dynamics seem to be significantly different in SZ patients versus healthy controls during resting condition (15 out of 28 studies reported significant differences), but altered activity was sometimes reduced (e.g., in the prefrontal cortex) and not excessive, depending on neural network location and condition. However, both local and global gamma activity seems to underlie disrupted neural connectivity-related symptoms in SZ [235]. Interestingly, recent findings imply a significant association between resting alpha activity and disease severity in SZ. The authors suggested that both low and high alpha activity are driven by different neural networks representing separate mechanisms for these sub-oscillations, with similar directional power differences (open versus closed eyes) found in the gamma frequency versus healthy controls [236]. Therefore, we suggest examining evoked or induced power (event-related EEG), which may reflect more sensitive biomarkers (e.g., early auditory ERP components) of functional decline and disease progression in SZ versus healthy controls, or versus other neuropsychiatric disorders [237]. Accordingly, other altered frequency-specific spectral power obtained via event-related EEG or MEG (magnetoencephalography) have demonstrated that adaptive control mechanisms in SZ are indexed by dysfunctional theta-band phase dynamics (that are phase-locked gamma activity during working memory activity), which are abnormally decoupled from frontal-cortex mechanisms that mediated cognitive control. Adaptive-control failures (affecting executive control mechanisms) in SZ are consistently supported by event-related theta activity abnormalities and the dysconnectivity hypothesis in SZ [238]. Specifically, theta oscillations are thought to enable flexible connections between different cortical networks mediating attentional control (within the prefrontal cortex) as attentional demands increase, allowing access to multiple memory networks. Functional and structural neural properties that perpetuate functional dysconnectivity in SZ often reflect hypoconnectivity across different frontal circuitries, independent of disease progression and duration. The genetic basis of altered PFC functional theta-connectivity in SZ is sparse. Functional theta connectivity that is associated with SZ risk genes should be further explored in SZ patients and their unaffected siblings, from prodrome periods to disease onset, and over recovery. In support of theta activity neural generators, the anterior cingulate cortex and the medial frontal cortex (MFG) and inferior parietal lobe areas seem to directly affect theta power modulations that are important for interference control in humans. In SZ, the magnitude of theta power changes is significantly lower in SZ versus healthy controls during the presentation of incongruent words [238,239]. Treatments targeting these areas that alter theta connectivity in high-risk individuals could impact the disease progression trajectory and may even suppress the severity of devastating positive symptoms at disease onset. Importantly, since SZ is associated with downregulation of brain-derived neurotrophic factor (*BDNF*), affecting neural plasticity and theta-related connectivity during increased attentional control conditions [238], it may be prudent to further examine the relationship between neural plasticity biomarkers in SZ (e.g., GABA-related risk genes, BDNF expression), event-related theta activity, and resting alpha/gamma activity with disease onset, severity, progression, treatment resistance, and functional outcome over time, in patients at high risk for SZ or in chronic SZ patients.

As such, an important avenue for future research will be to link microscale genomic transcriptional activity and proteomic expression with macroscale brain development and function. The tracking of temporal and spatial expression of risk genes that may produce multiple outcomes, such as SZ, autism, bipolar disorder, depression, epilepsy, and more, is most probably associated with the “when and where of their faulty expression or lack thereof. Differing developmental trajectories of gene expression will allow researchers to better understand why the same gene or groups of genes may pose a risk factor for more than one manifestation of illness [240].

Another direction to be explored is the study genes associated with SZ or other forms of mental illness but have not yet been studied in connection with neural oscillations. As an example, the *CACNA1A* gene, important for calcium channel function and neural communication, is associated with a broad spectrum of neurological disorders, but has not yet been linked to SZ or neural oscillations [241]. *SNX29*, involved in nervous system development, is a risk gene for several neurological disorders, including SZ and bipolar disorder, but its relation to neural oscillations has not yet been studied [242]. Another promising direction is the role of inflammation and its effects on GABAergic interneuron function. As cytokines such as Interleukin-1 beta, Tumor Necrosis Factor, and Interferon gamma are known to affect GABAergic interneuron function, it is hypothesized that the overexpression of these cytokines and others will directly affect neural oscillatory circuits. It will also be clinically productive to study the synergetic effects of cytokines with the panel of SZ risk genes. Notably, it has been suggested that IL-6 neuromodulation impacts glutamate and GABA receptor compartmentalization, which may significantly alter excitatory neurotransmission and lead to NMDA-induced excitotoxicity [243].

## Figures and Tables

**Table 1:** Altered genes, neural frequency-bands, and function in schizophrenia.

Gene	Frequency Band	Function	References
*GRIK3 (KAR)*	Gamma	KAR antagonists such as ACET reduce abnormal gamma; linked to SZ behavioral deficits	[33,34,35,36,37,38,39,40]
*KCNC1/2*	Gamma	Kv3.1/3.2 channels support FS interneuron function and gamma synchronization	[48,49,50,51,52,53,54,55,56]
*NRG1/ERBB4*	Gamma	*NRG1–ERBB4* interaction modulates GABA interneurons and fast oscillations	[61,62,63,64,65,66,67,68,69,70,71,72,73,74,75,76,77,78,79,80,81]
*NARP (MT-ATP6)*	Gamma	NARP regulates excitatory input to PV+ interneurons; deficits reduce gamma	[78,79,80,81,82,83,84]
*PLCB1*	Beta, Gamma, Theta	*PLCB1* knockouts impair theta/gamma and increase beta; endophenotypes ameliorated by clozapine	[85,86,87,88,89,90,91,92,93,168,169]
*GRM5*	Theta	mGluR5-NMDAR synergy enhances cognitive and theta-related function	[94,95,96,97,98]
*ARX*	Gamma, Theta	*ARX* regulates inhibitory neuron development; modulates evoked oscillations	[113]
*GRIN2A*	Gamma, Delta	*GRIN2A* KO mice show increased delta and disrupted gamma; associated with working memory deficits	[143,144,145,146,147,148,149]
*AKAP11*	Gamma	*AKAP11* mutation leads to abnormal gamma; linked to PKA pathway disruption	[150,151,160,161]
*GABRA2*	Beta (also alpha, gamma)	Downregulation of *GABRA2* linked to beta/gamma dysregulation in SZ	[162,163,164,165,166,167]
*ZNF804A*	Theta	Disrupts PFC–hippocampal theta coherence; affects memory networks	[171,172,173,174,175,176]
*COMT*	Theta, Delta, Beta, Alpha	*COMT* polymorphisms modulate prefrontal dopaminergic tone and oscillatory patterns	[177,178,179,180,181,182,183,184,185,186,187,188,189,190]
*KCNJ3/6/9/5*	Theta, Gamma	GIRK channels modulate hippocampal and thalamic theta/gamma oscillations	[191,192,193]
*CHRM1*	Gamma, Theta	*CHRM1* signaling affects memory and attentional oscillatory rhythms	[194]
*CACNA1I*	Delta	*CACNA1I* mutations reduce thalamic delta and sleep spindles	[200,201,202]
*SCN1A*	Delta, Gamma	*SCN1A* affects PV interneuron excitability; linked to abnormal delta/gamma activity	[203,204]
*HCN1*	Delta	*HCN1* channels regulate cortical delta rhythms via excitability control	[206,207,208,209,210,211,212]
*KCNB1*	Delta	*KCNB1* regulates AHP; implicated in delta abnormalities in SZ	[214,215,216,217,218]
*DISC1*	Theta, Gamma	*DISC1* regulates PV-IN dynamics and gamma synchronization	[219,220,221,222]
*NRGN*	Delta, Beta	*NRGN* affects synaptic plasticity and resting delta/beta coherence	[223,224,225,226,227]
*GNL3*	Alpha	*GNL3* linked to EEG alpha variation via GWAS data	[228,229,230]
*ITIH4*	Alpha	*ITIH4* associated with alpha rhythm modulation in postmortem SZ samples	[231,232]
*DTNPB1*	Gamma	Dysbindin intracelluualr protein trafficking and synaptic function	[114]
*PVALB*	Gamma	Parvalbumin calcium-binding protein expressed in GABAergic interneurons	[115]
*GAD1*	Delta, Theta, Alpha, Gamma	catalyzes the production of GABA from glutamate	[116,117,118]
*BDNF*	Alpha, Gamma	differentiation of neurons in the brain and spinal cord.	[119,120,121,122]

## Supplementary Materials

The following supporting information can be downloaded at: https://www.mdpi.com/article/10.3390/ijms26157514/s1.
