# Supplementary material for: Common Genomic and Proteomic Alterations Related to Disturbed Neural Oscillatory Activity in Schizophrenia
Source: Int J Mol Sci. 2025 Aug 4;26(15):7514. doi: 10.3390/ijms26157514 (PMC12347343; doi:10.3390/ijms26157514)

Supplementary Materials

Schematic representations of key signaling pathways

1. Phase Synchronization in GABAergic Interneurons

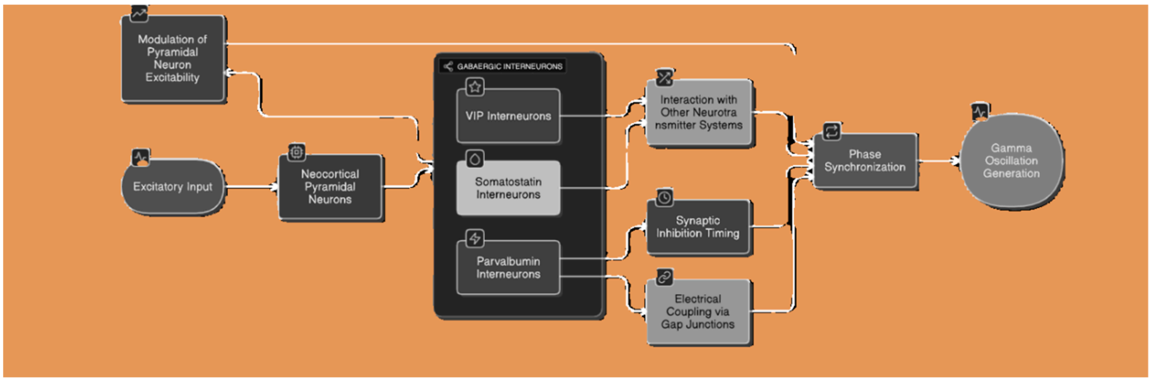

2. PLC-beta1 signaling pathways

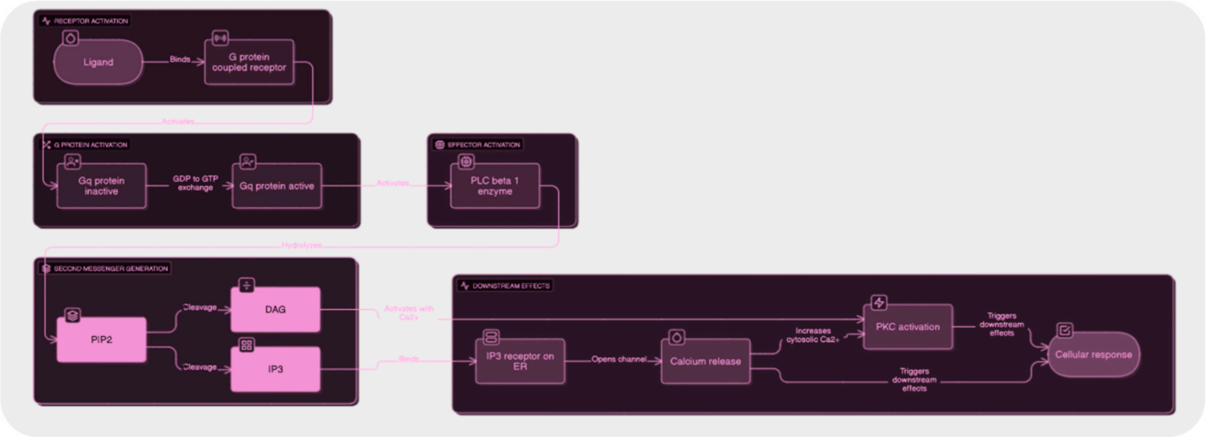

3. Neuregulin1 - ErbB4 signaling pathways

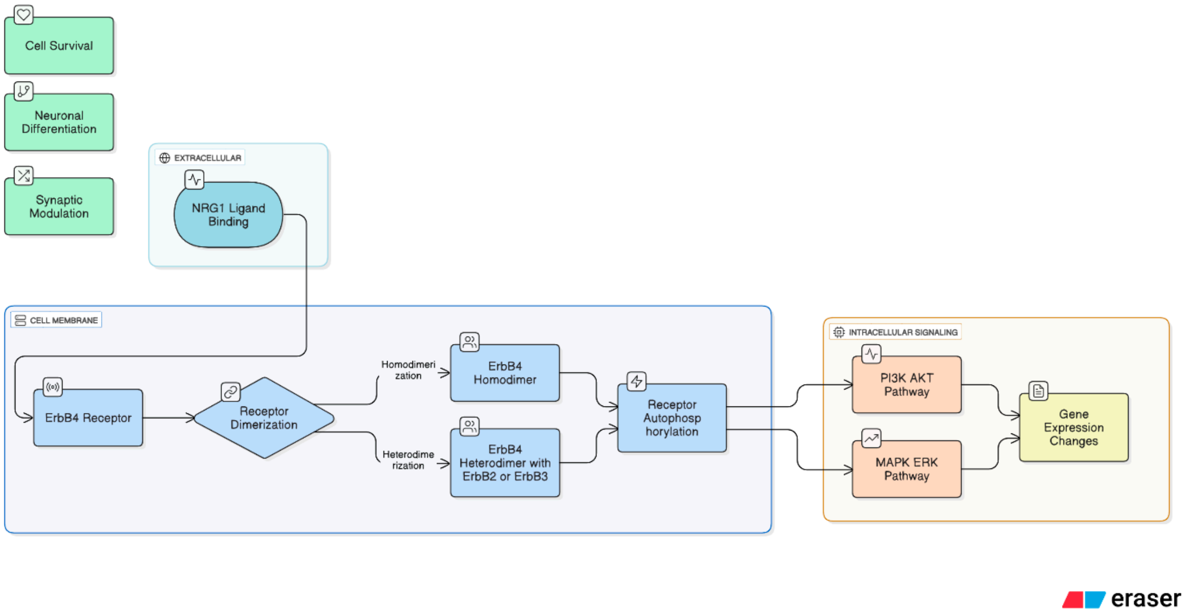

Supplement: Supplementary file 1 [file ijms-26-07514-s001.zip › ijms-3702620-supplementary.pdf]
